# Constitutive programmed death ligand 1 expression protects gastric G‐cells from *Helicobacter pylori*–induced inflammation

**DOI:** 10.1111/hel.12917

**Published:** 2022-07-28

**Authors:** Michiel C. Mommersteeg, Bing Ting Yu, Thierry P. P. van den Bosch, Johan H. von der Thüsen, Ernst J. Kuipers, Michael Doukas, Manon C. W. Spaander, Maikel P. Peppelenbosch, Gwenny M. Fuhler

**Affiliations:** ^1^ Department of Gastroenterology and Hepatology Erasmus MC University Medical Center Rotterdam The Netherlands; ^2^ Department of Pathology Erasmus MC University Medical Center Rotterdam The Netherlands

**Keywords:** intestinal metaplasia, gastric cancer, *Helicobacter pylori*, PD‐L1, gastrin

## Abstract

**Introduction:**

Gastric intestinal metaplasia (GIM) is a premalignant lesion, highly associated with *Helicobacter pylori* infection. Previous studies have shown that *H. pylori* is able to induce the expression of programmed death ligand 1 (PD‐L1), an inhibitory immune modulator, in gastric cells. Our aim was to investigate whether tissues from GIM patients may exploit PD‐L1 expression upon *H. pylori* infection to evade immunosurveillance.

**Methods:**

Immunohistochemistry was performed for PD‐L1 and enteroendocrine markers somatostatin and gastrin on samples derived from a cohort of patients with known GIM, both before and after *H. pylori* eradication. To determine the identity of any observed PD‐L1‐positive cells, we performed multiplex immunofluorescent staining and analysis of single‐cell sequencing data.

**Results:**

GIM tissue was rarely positive for PD‐L1. In normal glands from GIM patients, PD‐L1 was mainly expressed by gastrin‐positive G‐cells. While the D‐cell and G‐cell compartments were both diminished 2‐fold (*p* = .015 and *p* = .01, respectively) during *H. pylori* infection in the normal antral tissue of GIM patients, they were restored 1 year after eradication. The total number of PD‐L1‐positive cells was not affected by *H. pylori*, but the percentage of PD‐L1‐positive G‐cells was 30% higher in infected subjects (*p* = .011), suggesting that these cells are preferentially rescued from destruction.

**Conclusions:**

Antral G‐cells frequently express PD‐L1 during homeostasis. G‐cells seem to be protected from *H. pylori*‐induced immune destruction by PD‐L1 expression. GIM itself does not express PD‐L1 and is unlikely to escape immunosurveillance via expression of PD‐L1.

**Synopsis (statement of significance)**

In this manuscript, we show selective and constitutive expression of PD‐L1 expression on G‐cells of the stomach, which through their immunomodulatory effects may protect these enteroendocrine cells from *H. pylori*‐mediated inflammatory destruction. This is the first‐time constitutive expression of PD‐L1 shown in a single gastric cell type and in cells of the enteroendocrine lineage. Furthermore, we show enteroendocrine cell compartments are restored after *H. pylori* has been eradicated even in patients who have already developed gastric premalignant lesions.

## 1 INTRODUCTION

*Helicobacter pylori* (*H. pylori*) colonizes half of the world's population. This gram‐negative, rod‐shaped bacterium is of particular interest as it is the only bacterium thus far recognized as a class one carcinogen.^1^ When *H. pylori* colonizes the stomach, it sets in motion a cascade of events starting with chronic inflammation that may eventually lead to mucosal trans‐differentiation into an intestinal phenotype. This phenomenon is called gastric intestinal metaplasia (GIM) and is thought to be a precursor stage to gastric cancer (GC).^2^ To date there are no curative options for GIM, therefore surveillance of these lesions is often implemented, as early detection of GC may improve the 5‐year overall survival from an abysmal 25% to 90%.^3^, ^4^

Although the association between *H. pylori*, GIM, and GC is clear, the exact mechanisms governing the carcinogenic process are still largely unknown. Disturbances in the enteroendocrine cell compartments regulating gastric acidity have been implicated in GC development.^5^ Acid production by parietal cells is stimulated through histamine released by enterochromaffin‐like cells (ECL‐cells), which in turn are activated by gastrin produced by G‐cells. Inhibition of G‐cells, ECL‐cells, and parietal cells is established through somatostatin released by D‐cells. Aside from maintaining homeostasis of the gastric acidic environment, gastrin is also an important growth factor for the stomach epithelium, and the entire gastrointestinal tract.^6^, ^7^ Gastrin achieves these trophic effects through binding with the cholecystokinin 2 receptor (CCK‐2R), which subsequently results in the expression of several growth factors. These include amphiregulin epidermal growth factor and Sonic Hedgehog, both known to play a role in carcinogenesis.^5^, ^8^ Conversely, more and more evidence suggests that somatostatin, a gastrin antagonist, has potent antitumor effects, with the somatostatin encoding gene *SST* considered to be a tumor suppressor susceptible to epigenetic silencing in several cancers.^4^, ^5^, ^9^, ^10^ Reports have indicated that *H. pylori* may cause the destruction of D‐cells,^11^ thereby leading to enhanced gastrin production. This has been suggested as a potential mechanism contributing to *H. pylori*‐induced carcinogenesis. Increased numbers of G‐cells or upregulated G‐cell activity in *H. pylori*‐infected individuals and in the development of GC has also been reported, but this remains disputed.^11^, ^12^, ^13^, ^14^, ^15^, ^16^, ^17^

With GC being such a lethal disease, efforts to implement alternative treatment strategies are ongoing. Over the last couple of years, the success of immune checkpoint inhibitors in the treatment of several tumor types spiked interest in their use for GC as well. Immune checkpoint receptors provide co‐inhibitory signals that the tumor uses to escape immunosurveillance.^18^, ^19^ One of the best‐described checkpoint inhibitor pathways is the programmed death 1/programmed death ligand 1 (PD‐1/PD‐L1) pathway. Many tumors express PD‐L1, which inhibits the activation and proliferation of cytotoxic T‐cells expressing PD‐1. Blocking these co‐inhibitory signals allows the immune system to help destroy the tumor. This approach has been very successful in improving survival and changing treatment paradigms in, for instance melanoma and non‐small‐cell lung cancer.^20^, ^21^ Clinical trials have reported positive results for PD‐1 inhibitors in GC as well,^22^ although the response rate appears to be relatively low, and limited to patients with microsatellite instability.^23^ Interestingly, a recent study showed that patients with gastric spasmolytic polypeptide expressing metaplasia (SPEM) expressed PD‐L1 in the presence of *H. pylori*.^24^ These data confirm several older studies, which demonstrated that PD‐L1 protein expression was increased in cell lines and mice in the presence of *H. pylori*. Taken together, these studies suggest that *H. pylori* may limit the clearance of SPEM cells in the gastric mucosa through upregulation of checkpoint inhibition molecules, thereby promoting the carcinogenic cascade.^25^, ^26^ Our primary aim was to investigate whether PD‐L1 expression is also present in the mucosa of gastric GIM patients (thought to be a subsequent step to SPEM in the premalignant cascade), and to what extent this is influenced by the presence of *H. pylori*. We hypothesized that PD‐L1 expression in GIM might allow escape from immunosurveillance and subsequent progression towards cancer. Surprisingly, we find that PD‐L1‐expressing cells are indeed present in gastric mucosa from GIM patients, but only in normal gastric glands. Furthermore, while our data do not show an upregulation of PD‐L1 in gastric tissue from *H. pylori*‐positive GIM patients, they do support the notion that PD‐L1 may protect G‐cells from *H. pylori*‐induced destruction, thereby potentially contributing to proliferative signals.

## 2 MATERIAL AND METHODS

### 2.1 Patient material

Gastric biopsies came from participating patients in a multicenter prospective cohort study on gastric premalignant lesions; the Proregal study.^27^, ^28^ Patients who had pathology‐proven GIM at their index (t0) endoscopy were eligible for inclusion in this study. If *H. pylori* was discovered at or around the index endoscopy it was eradicated successfully in all patients. The first follow‐up time point was planned 1 year after the index endoscopy, and the second follow‐up time point was planned 3 years after the index endoscopy. Thereafter, the follow‐up was planned in accordance with the MAPS guidelines.^4^ At each follow‐up time point, a complete mapping of the stomach was performed. Biopsies were taken from every compartment of the stomach: four from the antrum, two from the angulus, two from the greater curvature of the corpus, two from the lesser curvature of the corpus, and two from the cardia. Biopsies were formalin‐fixed and embedded in paraffin and slides were prepared with a thickness of 4 μm. Patient characteristics can be found in Table 1. The study protocol was approved by the Institutional Review Board (MEC‐2009‐090), and all patients signed an informed consent.

**TABLE 1:** Baseline characteristics

Baseline characteristics	
Age at inclusion, mean (SD)	57.6 (11.7)
Sex, male (%)	27 (56.3%)
Ethnicity, Caucasian (%)	39 (81.3%)
*H. pylori*, positive (%)	24 (50%)
OLGIM 0 (%)	1 (2.2%)
OLGIM I (%)	9 (18.8%)
OLGIM II (%)	20 (41.7%)
OLGIM III (%)	11 (22.9%)
OLGIM IV (%)	4 (8.3%)

### 2.2 Immunohistochemistry

Briefly, 4 μm sections were deparaffinized in xylene twice and rehydrated through graded ethanol 100% ethanol twice, 96% solution ethanol, and 70% solution ethanol. Slides were rinsed several times with fresh deionized water, followed by one wash with tap water. Heat‐induced epitope retrieval was performed by using 10 mM sodium citrate buffer (pH 6.0) for 15 min. After epitope retrieval, slides were cooled slowly for 45 min before being washed three times for 5 min in PBS. Endogenous peroxides were blocked by PBS/3% H_2_O_2_ solution for 10 min at room temperature (RT). Slides were washed with PBS and blocked by 10% normal goat serum in PBS for 1 h at RT. Afterwards, the primary antibody was added as described in Table S1 and incubated overnight at 4°C. Slides were washed with PBS. Rabbit envision (DAKO) was added as a secondary antibody and incubated for 30 min at RT. Visualization was performed by using Tris/HCL buffer (pH 7.6) containing 0.03% H_2_O_2_ and 0.5 mg/ml di‐amino‐benzidine (DAB), incubated for 10 min. Slides were counterstained with hematoxylin, washed with tap water, and dehydrated using 70% solution of ethanol, then 96% solution of ethanol and twice 100% ethanol, and finally twice in xylene. Slides were mounted with Pertex and a coverglass. A minimum of four images were taken from each slide at 20× magnification. For control staining images, see Figure S1.

### 2.3 Multiplex immunofluorescent staining

To determine the identity of the PD‐L1‐positive cells, triple stains were performed by automated multiplex immunofluorescence using the Ventana Benchmark Discovery (Ventana Medical Systems Inc.). In brief, following deparaffinization and heat‐induced antigen retrieval with CC1 (#950‐500, Ventana) for 64 min at 97°C, the tissue samples were incubated firstly with antibodies against either CD45, gastrin, chromogranin‐A, or somatostatin for 32 min at 37°C followed by detection with either Ultramap anti‐rabbit HRP (#760‐4315, Ventana) or Ultramap anti‐mouse HRP (#760‐4313, Ventana) for 12 min, followed by visualization with Red610 for 8 min (#760‐245, Ventana). Antibody denaturing was performed using CC2 (#950‐123, Ventana) for 20 min at 100°C. Secondly, PDL1 SP263 was incubated for 32 min at 37°C followed by detection with Ultramap anti‐rabbit HRP (#760‐4315, Ventana), followed by visualization with FAM (#760‐243, Ventana) for 4 min. Slides were incubated in PBS with DAPI for 15 min and covered with an anti‐fading medium (DAKO, S3023). Antibody information can be found in Table S1.

### 2.4 Analysis of publicly available single‐cell sequencing data

Data were acquired from the publication of Zhang et al^29^ in Cell reports ‘Dissecting the Single‐Cell Transcriptome Network Underlying Gastric Premalignant Lesions and Early Gastric Cancer’. Clustering was performed using R (4.0.2) Seurat package. The Seurat object was devised from GSM3954946, GSM3954947, GSM3954948, GSM3954949, GSM3954950, GSM3954951, GSM3954952, GSM3954953, GSM3954954, GSM3954955, GSM3954956, GSM3954957 via the CreateSeuratObject function (settings: min.cells = 3, min.features = 200). If they met one of the following thresholds, cells would be flagged as poor quality and excluded: (1) The number of genes was expressed below 400 or above 7000; (2) the mapping of mitochondrial or ribosomal genes was 20% or more of the unique molecular identifiers. In the single‐cell data, we identified G‐cells and D‐cells by the following markers: ‘*GAST*’, ‘*CHGA*’, ‘*CHGB*’, and ‘*SST*’, ‘*CHGA*’, ‘*CHGB*’ genes, respectively. The marker genes for other cell types can be found in Table S2, with visualization in Figure S2.

### 2.5 Statistical analysis

Normality testing was performed using the Shapiro–Wilk tests. Statistical significance was determined using student's *t*‐test (two‐tailed *p*‐values) for two‐way comparisons of normally distributed data, two‐way comparisons of non‐normally distributed data were performed using the Mann–Whitney test (nonpaired) or Wilcoxon test (paired samples). For multiple comparisons, one‐way ANOVA was used with post hoc multiple comparisons correction using Tukey. Statistical analysis was performed using the statistical package GraphPad Prism version 8.0.2 (GraphPad Software Inc.). A two‐sided *p‐*value of less than .05 was considered statistically significant while values less than .1 indicated a trend.

## 3 RESULTS

### 3.1 PD‐L1 expression is present in normal antral glands in GIM patients, but not in GIM tissue

**FIGURE 1:**
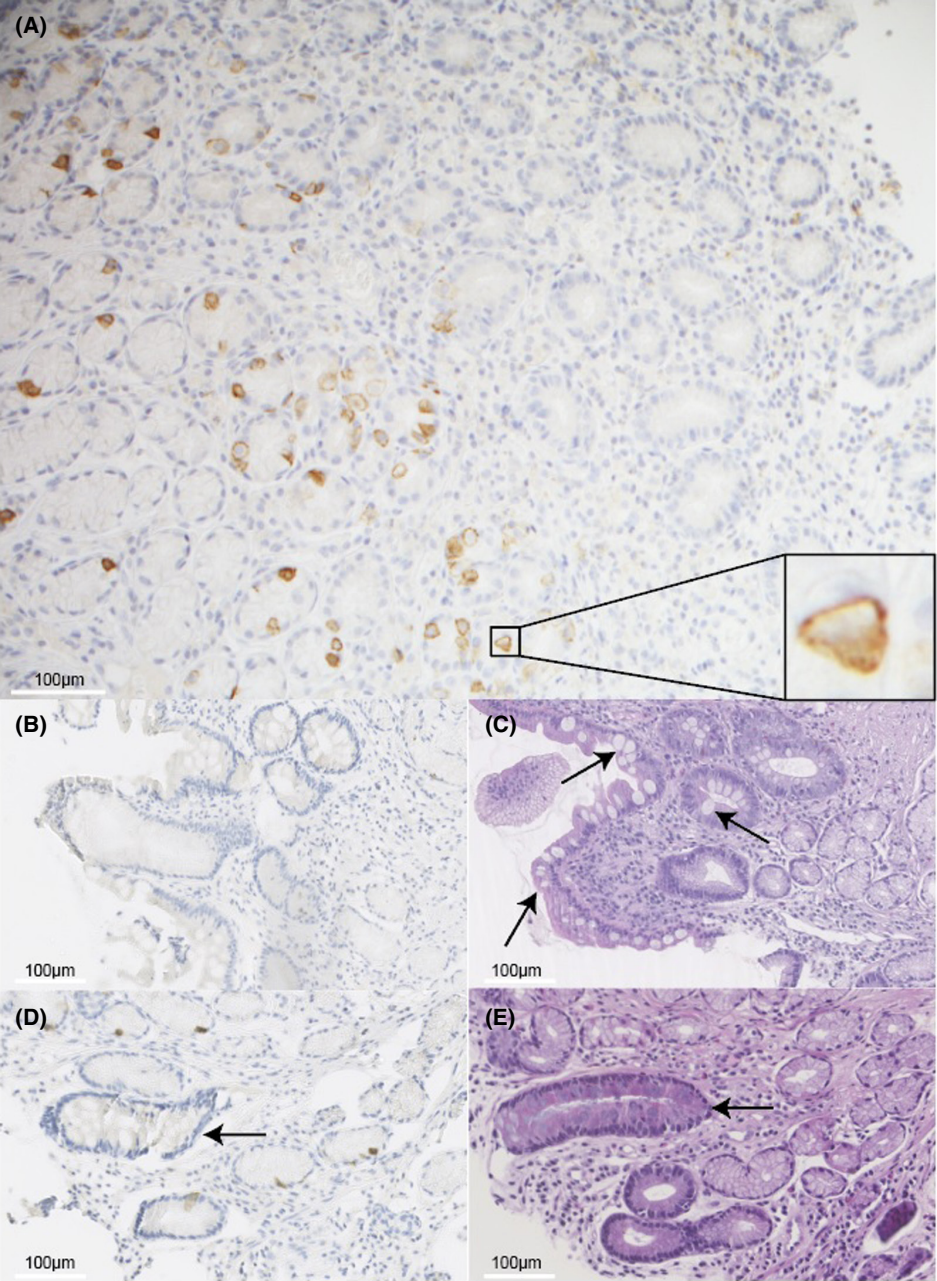
PDL‐1‐positive cells are present in gastric mucosa from intestinal metaplasia (IM) patients, but not in metaplastic glands. (A) Representative images of antral biopsies immunohistochemically stained for PD‐L1 showing that while normal antral epithelium contains numerous PD‐L1‐positive cells in the glandular layer of the epithelium (brown staining), intestinal metaplastic glands of the antrum show no PD‐L1‐positive cells. PD‐L1 staining (B) and matched hematoxylin–eosin (H&E, C) stain of antrum with intestinal metaplastic crypts. Intestinal metaplasia is recognized by the presence of goblet cells (arrows). PD‐L1 staining (D) and matched H&E stain (E) of antrum showing no PD‐L1 expression in metaplastic crypts (arrow), while in neighboring normal glands, a subpopulation of glandular cells does express PD‐L1.

First, we investigated whether PD‐L1 expression was present in biopsies from patients with GIM. As shown in Figure 1A, a subpopulation of gastric epithelial cells staining positive for PD‐L1 was indeed present in mucosal tissue from GIM patients. However, when investigating whether PD‐L1 was expressed in intestinal metaplastic glands, we found almost no cells positive for PD‐L1 in glands with intestinal‐type goblet cells (Figure 1B,C), while adjacent normal glands did contain PD‐L1‐positive cells (Figure 1D,E). In all biopsies studied (*n* = 30), only 2 PD‐L1‐positive cells were detected in intestinal metaplastic glands making it unlikely that PD‐L1 expression protects GIM glands from immunosurveillance. Indeed, PD‐L1 positivity did not correlate with progression or regression of OLGIM stage in this small cohort of patients (Figure S3).

**FIGURE 2:**
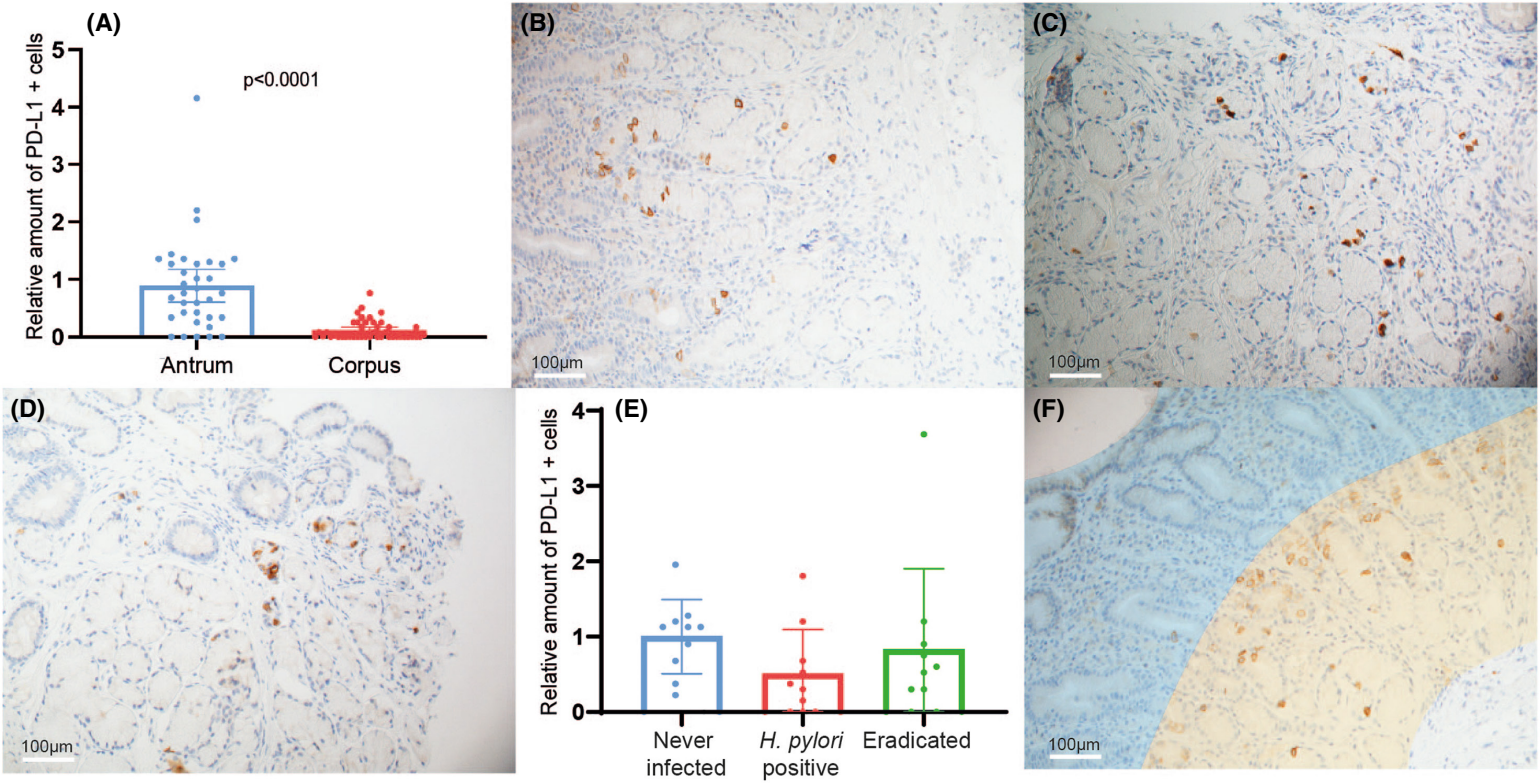
PD‐L1 expression is present in the glandular layer just below the foveola in normal antral crypts, and not affected by *H. pylori* infection. (A) Quantification of the relative number of PD‐L1‐positive cells of the corpus (*n* = 44) as compared to the antrum (*n* = 34), indicating a lower abundance of PD‐L1‐positive cells in the corpus of the stomach. Significance was calculated using the Wilcoxon test. Representative images of antral biopsies immunohistochemically stained for PD‐L1. (B) Antral biopsy of a patient actively infected with *H. pylori* with no intestinal metaplasia present in this biopsy. (C) Antral biopsy of a patient negative for *H. pylori* after eradication, no intestinal metaplasia present in this biopsy. (D) Antral biopsy of a patient never infected with *H. pylori*, with intestinal metaplasia present in the upper left quadrant of this biopsy. (E) Comparison of the relative number of PD‐L1‐positive cells between patients actively infected with *H. pylori* (*n* = 10) and these same patients after eradication, compared against the mean number of PD‐L1‐positive cells of patients who were never tested positive for *H. pylori* (*n* = 13). Significance was calculated using an ANOVA test, post hoc testing was not performed as the ANOVA showed no significant difference. (F) Picture of antral biopsy immunohistochemically stained for PD‐L1 with an overlay showing the superficial foveolar epithelium in blue and the glandular epithelium in orange.

We found a significantly higher number of PD‐L1‐positive cells in antrum biopsies as compared to corpus biopsies of the same GIM patients (Figure 2A). As previous research has shown that *H. pylori* induces PD‐L1 protein expression in gastric epithelial cells and GIM is known to be an inhospitable environment for *H. pylori*,^24^, ^25^, ^30^ we next asked whether *H. pylori* infection influences antral PD‐L1 expression. PD‐L1 immunohistochemistry was performed on biopsies of patients during *H. pylori* infection (Figure 2B) and at least 1 year after successful eradication in the same individuals (Figure 2C). Patients diagnosed with intestinal metaplasia but with negative *H. pylori* serology, breath test, and histology were used as controls (Figure 2D). No significant difference was seen when comparing the number of PD‐L1‐positive cells in *H. pylori*‐positive patients before or after treatment compared with never‐infected individuals (Figure 2E), suggesting that *H. pylori* infection does not influence the number of PD‐L1‐positive cells in the gastric antrum.

### 3.2 PD‐L1 positivity in the gastric epithelium co‐localizes with chromogranin‐A and Gastrin

**FIGURE 3:**
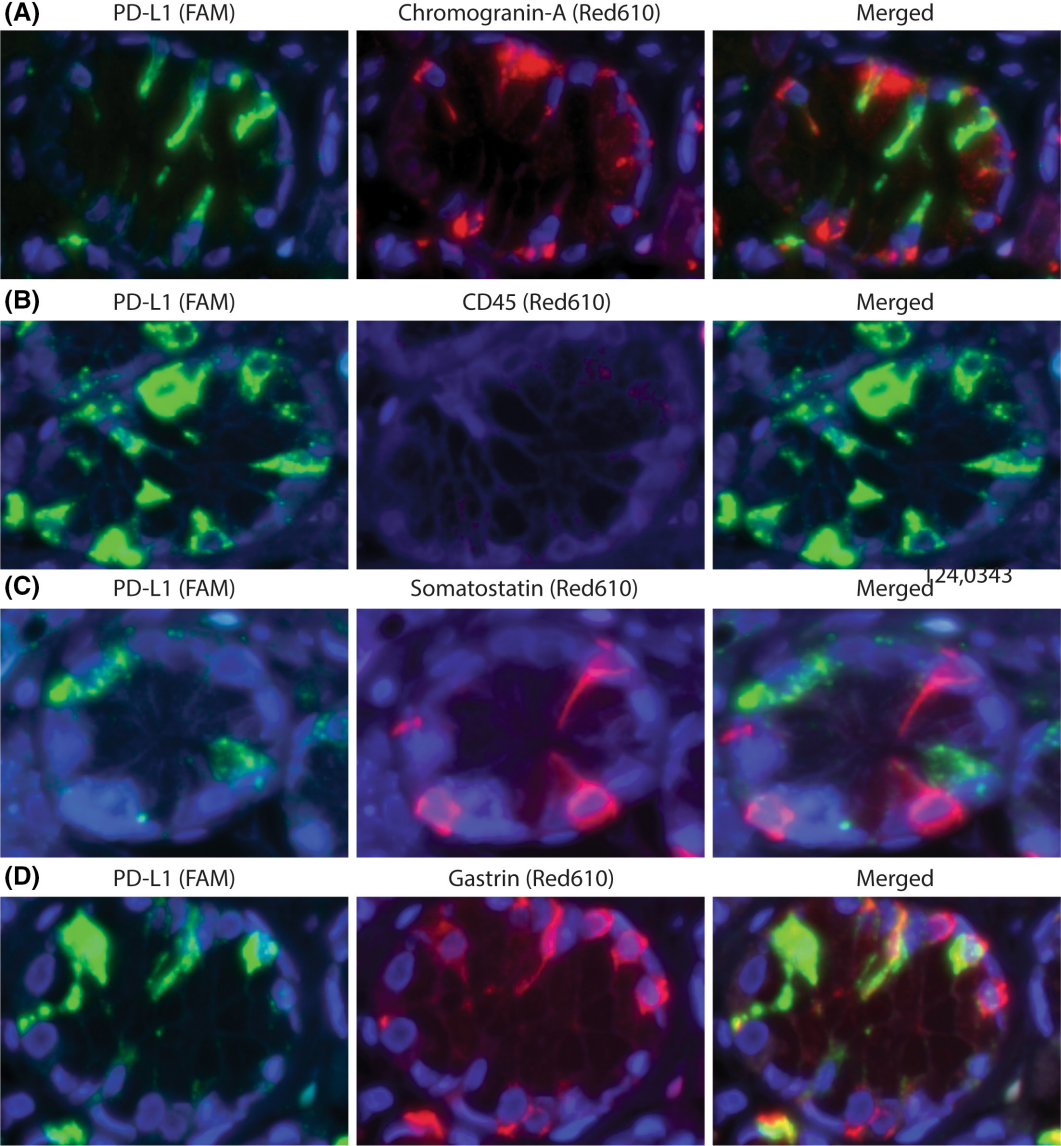
PD‐L1 is expressed in gastrin‐positive cells but not in CD45‐ or somatostatin‐positive cells. Representative multiplex immunofluorescence images from single antral glandular crypts. (A) PD‐L1 (green) co‐localizes with chromogranin‐A (red). (B) CD45 (red) does not co‐localize with epithelial PD‐L1 (green). See also Figure S5. (C) Somatostatin (red) does not co‐localize with PD‐L1 (green). (D) Gastrin (red) does co‐localize with PD‐L1 (green).

The above rather unexpected findings led us to further investigate the nature of the PD‐L1‐positive cells in the unaffected gastric mucosa of GIM patients. PD‐L1‐positive cells were predominantly present in the antrum, and located just below the isthmus of the antral glands, within the region containing the transition from foveolar cells to the glandular epithelium (Figure 2F). Above the isthmus, almost no PD‐L1‐positive cells were found. Furthermore, PD‐L1‐positive cells seemed to be a part of the gastric epithelium and not part of the lamina propria or infiltrating immune cells. Taken together this location suggests an enteroendocrine origin of these PD‐L1‐positive cells.^31^ To confirm this, we performed multiplex immunofluorescence staining for PD‐L1 and the common gastrointestinal enteroendocrine cell marker chromogranin‐A. As can be appreciated in Figure 3A, chromogranin‐A indeed co‐localized with PD‐L1 in antral glands from GIM patients (more images shown in the Figure S4). To exclude the possibility that we were observing immune cells (i.e., infiltrating macrophages), we also performed immunofluorescence double staining for PD‐L1 and CD45 (Figures 3B and S5), which did not demonstrate any co‐localization of these two markers in the gastric epithelial compartment.

Three different types of enteroendocrine cells can be identified in the gastric antral mucosa: D‐cells, G‐cells, and ECL‐cells.^31^ To determine which of these cell populations may be PD‐L1‐positive, we first performed multiplex immunofluorescence staining for PD‐L1 and somatostatin, representing the D‐cell population. No cells double positive for these two markers were found in any of the glands analyzed (Figure 3C). Next, we performed multiplex immunofluorescence for PD‐L1 and gastrin as a measure of G‐cells. PD‐L1 clearly co‐localized with gastrin‐positive cells within the same gland (Figures 3D and S6). Upon quantification of the total number of cells from 12 biopsies, half (54.8%) of the cells within the G‐cell compartment were found to be positive for PD‐L1 (Table 2). Conversely, less than 5% of the total PD‐L1‐positive epithelial cells lacked gastrin expression, indicating that PD‐L1 positivity in gastric biopsies is mainly restricted to G‐cells. PD‐L1 expression and co‐localization to G‐cells were not specific to normal mucosa from GIM patients: Gastric sleeve tissue from stomach reduction surgery from a patient with an otherwise normal gastric antral mucosa showed comparable co‐localization of PD‐L1 and gastrin (Figure S7). We conclude that PD‐L1 positivity of G‐cells is independent of the presence of gastric premalignant lesions.

**TABLE 2:** Quantification of co‐localization of PD‐L1 and gastrin

	PD‐L1 + Gastrin +, % (IQR)		PD‐L1 − Gastrin +, % (IQR)		PD‐L1 + Gastrin −, % (IQR)	
Overall (*n* = 12)	54.7 (38.0)		40.7 (34.54)		4.6 (3.85)	
*H. pylori*‐infected (*n* = 5)	71.6 (16.1)	*p* = .011	22.2 (19.4)	*p* = .0084	6,2 (3.2)	ns
*H. pylori*‐eradicated (*n* = 6)	42.0 (22.9)		54.7 (26.5)		3.3 (3.7)	
Never‐infected (*n* = 1)	46.1		49.4		4.5	
Normal antral mucosa (*n* = 1)	36.5		58.1		5.4	

*Note*: During an active *H. pylori* infection, the percentage of PD‐L1/gastrin dual positive cells is significantly increased as compared to the same patients after eradication (*n* = 5). While the amount of gastrin single positive cells decreases during active *H. pylori* infection.

Abbreviations: IQR, interquartile range; PD‐L1, programmed death ligand 1.

**FIGURE 4:**
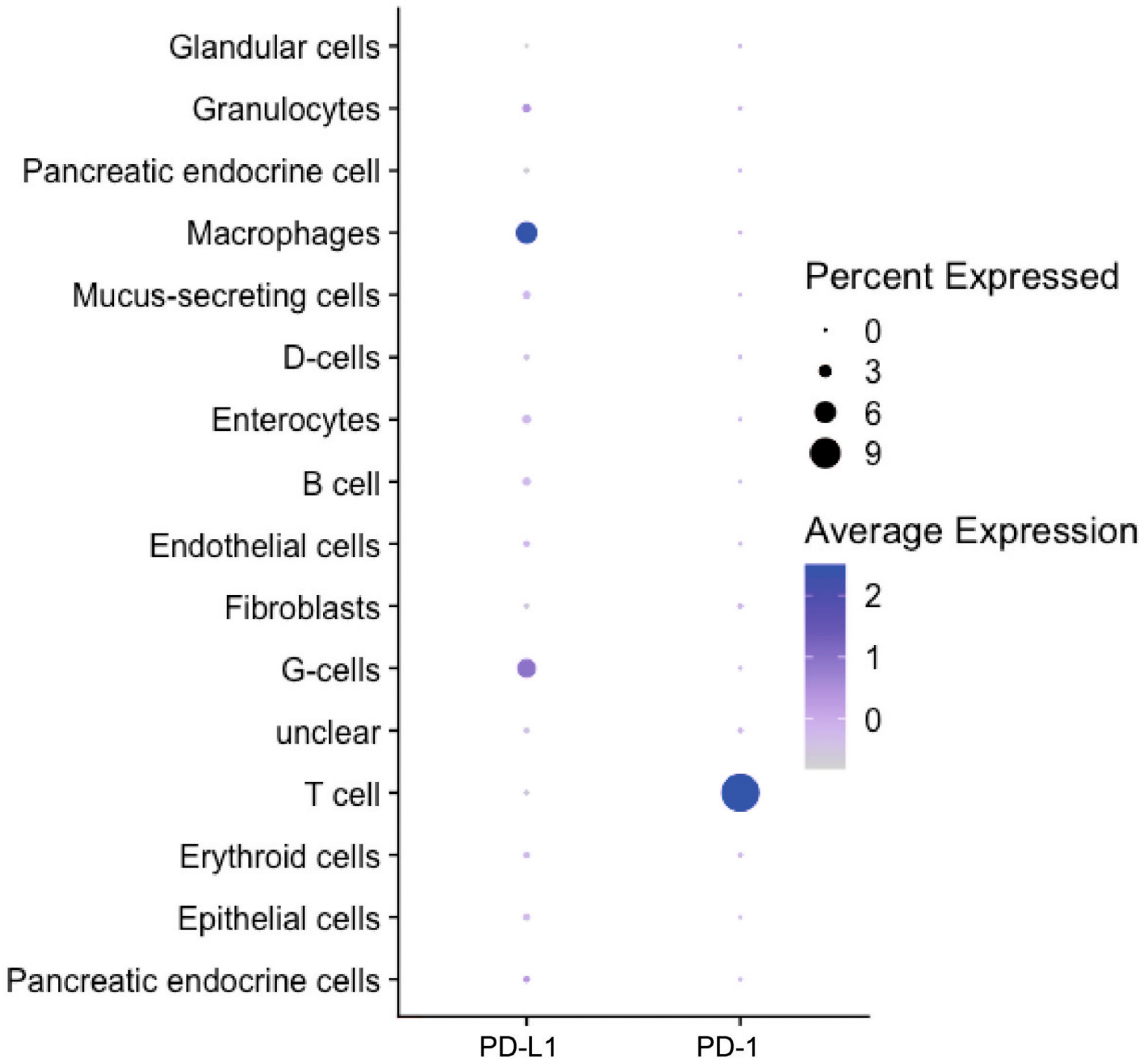
Single‐cell RNA‐sequencing data derived from publicly available data. Graphical representation of the percentage of cells expressing PD‐L1 or PD‐1 mRNA transcripts and the strength of expression within different cell clusters. All biopsies (*n* = 12) from all subjects were pooled in this analysis (25).

We further confirmed these data by investigating publicly available single‐cell sequencing data.^29^ The dataset comprised 12 biopsies from nine donors, two of whom were *H. pylori*‐infected and four of whom showed GIM. While PD‐L1 expression was generally low, the cell populations most associated with PD‐L1 expression were G‐cells and macrophages (Figure 4). As expected, the expression of the ligand PD‐1 in the gastric mucosa was limited to T‐cells.

### 3.3 Both D‐ and G‐cell compartments are diminished in the gastric antrum during *H. pylori* infection but are restored after eradication

**FIGURE 5:**
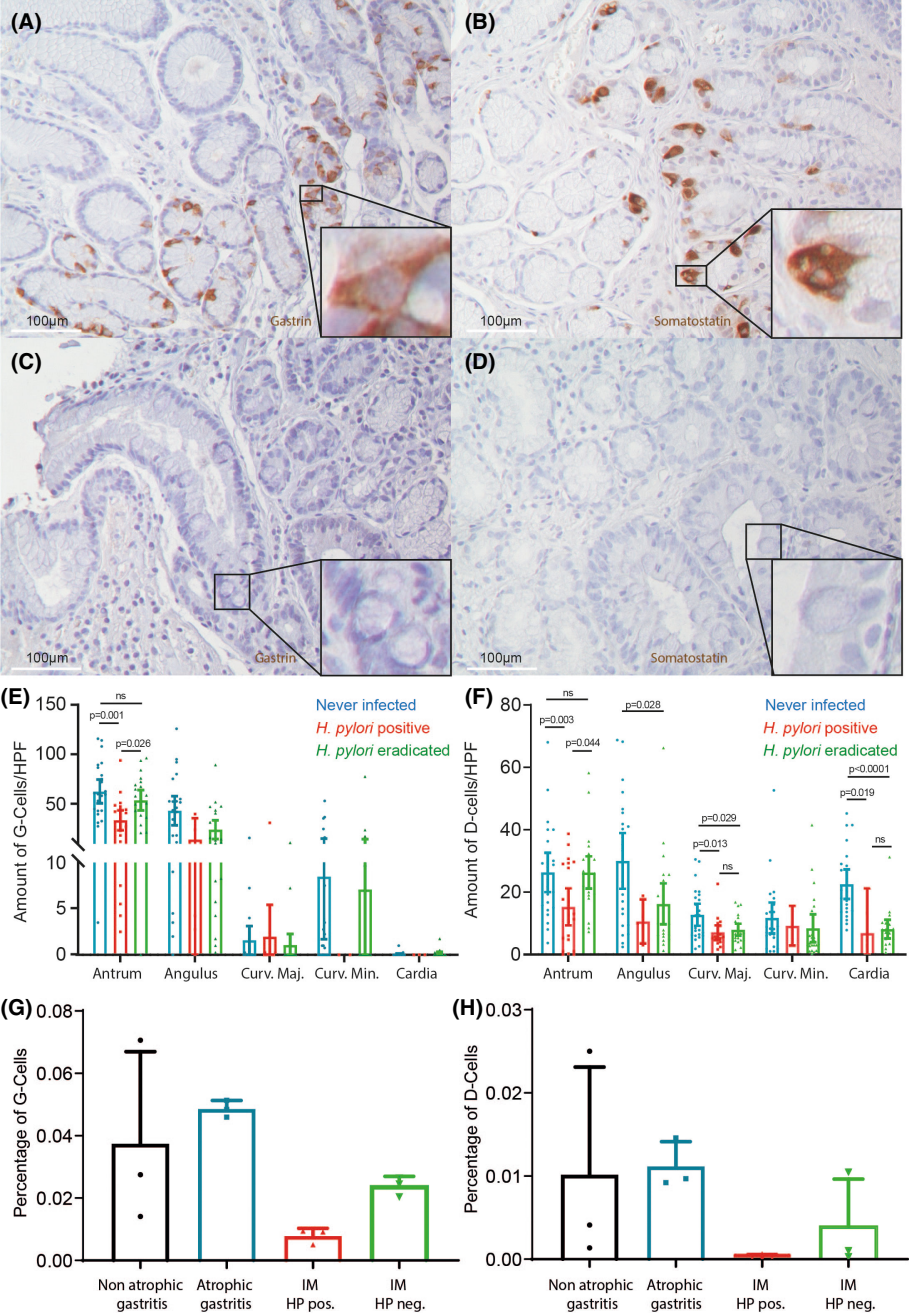
Both G‐ and D‐cell compartments are diminished during *H. pylori* infection and restored after eradication. Representative images of biopsies immunohistochemically stained for gastrin (A) and somatostatin (B). Representative images of biopsies in fields of GIM immunohistochemically stained for gastrin (C) and somatostatin (D). Quantification of the average number of gastrin‐positive cells (E) and somatostatin‐positive cells (F) per high power field, per stomach location compartment. Never‐infected (*n* = 24), *H. pylori‐*infected (*n* = 24), and *H. pylori*‐eradicated samples from these same individuals (*n* = 24) were included. (G) Quantification of the percentage cells identified as G‐cells of the total amount of cells following our clustering of publicly available data from single‐cell sequencing (*n* = 3 for each subgroup) (25). (H) Quantification of the percentage cells identified as D‐cells of the total cells following our clustering of publicly available data from single‐cell sequencing (*n* = 3 for each subgroup).

Previous studies suggested that *H. pylori* affects the presence of enteroendocrine cells.^14^, ^32^ To investigate to what extent this holds true in GIM patients, we investigated different regions of the stomach for the presence of gastrin and somatostatin to indicate G‐ and D‐ cells, respectively (examples shown in Figures 5A,B and S1). As might be expected, neither of these enteroendocrine cell compartments were present in GIM regions of the mucosa from GIM patients (examples shown in Figure 5C,D).^33^ Furthermore, quantification of gastrin staining indicates that, as expected, G‐cells are mainly limited to the normal antrum and angulus (Figure 5E). This supports the observation that no PD‐L1 expression (which is mainly limited to G‐cells) was seen in intestinal metaplastic parts of the antrum, nor in the corpus, of GIM patients. In GIM patients, *H. pylori* was associated with a marked decrease in the number of G‐cells in the antrum and angulus when compared to never‐infected individuals (*p* = .0005) and the same patients after they were cured from *H. pylori* infection (*p* = .0056, Figure 5E). After eradication of *H. pylori* the G‐ cell compartment was completely restored in the antrum, as there was no significant difference in the number of G‐cells in patients that were cured and those that have never been infected with *H. pylori*, while this was not the case for the angulus. The number of D‐cells, which are present throughout all compartments of the stomach, is also diminished during *H. pylori* infection when compared to never infected patients (*p* = .0057) and the same patients after they were cured from *H. pylori* infection (*p* = .0155; Figure 5F). Again, restoration of D‐cell numbers was seen upon eradication, but only in the antrum (Figure 5F). The same trend was observed in the publicly available single‐cell sequencing dataset discussed earlier^29^; *H. pylori‐*infected tissues containing GIM showed fewer cells that could be identified as either G‐cells or D‐cells, supporting the data, we found in our immunohistochemistry experiments (Figure 5G,H).

### 3.4 Within the G‐cell compartment the percentage of PD‐L1‐positive cells increases in *H. pylori* infections

Given the fact that PD‐L1 was exclusively observed on G‐cells in the normal mucosa from GIM patients (and not in the GIM parts of the tissue), we were surprised to find that the total number of these G‐cells diminished during *H. pylori* infection, while the total number of PD‐L1‐positive cells did not. Therefore, we investigated the percentage of PD‐L1‐positive cells within the G‐cell compartment during active *H. pylori* infection. Indeed, we found that during *H. pylori* infection, the percentage of PD‐L1‐positive G‐cells significantly increases within this compartment (*p* = .011, Table 2). This suggests that G‐cells use PD‐L1 to escape *H. pylori*‐induced T‐cell mediated inflammatory destruction during active infection.

## 4 DISCUSSION

To the best of our knowledge, this is the first study that shows constitutive PD‐L1 expression in the gastric G‐cell compartment of patients diagnosed with GIM. This PD‐L1 expression seems to be independent of the presence of both intestinal metaplasia and *H. pylori*. As intestinal metaplastic parts of the gastric mucosa do not contain any enteroendocrine cells, both PD‐L1 and the G‐cells on which this protein is expressed in GIM patients were limited to the normal glands in these patients. Furthermore, we observed PD‐L1‐positive G‐cells in the stomach tissue of a healthy individual, suggesting that this is a normal physiological phenomenon, although studies including more control samples will need to validate this. The lack of PD‐L1 in metaplastic glands from GIM patients suggests that progression of GIM to GC is unlikely to occur via protection of GIM glands from immunosurveillance through PD‐L1.

Increased or aberrant PD‐L1 expression in both epithelial compartments and immune cells has been demonstrated in several diseases, including several types of (gastrointestinal) cancers^34^, ^35^, ^36^, ^37^, ^38^, ^39^, ^40^, ^41^ and gastric adenocarcinomas.^42^ However, the timing within the carcinogenesis cascade at which this expression pattern starts to deviate is unclear. We show that GIM precursor lesions do not express PD‐L1. It was previously reported that SPEM cells, a subtype of intestinal metaplasia marked by an antral foveolar phenotype, upregulate PD‐L1 in the presence of *H. pylori*.^24^ It was postulated that these metaplastic cells use PD‐L1 expression to escape immunosurveillance induced by *H. pylori*‐associated inflammation. However, the lack of PD‐L1 expression in GIM glands seen in our study, even during *H. pylori* infection, suggests that its expression may be lost during the carcinogenic cascade. Indeed, PD‐L1 was mainly seen on G‐cells, which are no longer present upon formation of GIM. GIM and SPEM represent two separate identities, which may partly explain the differences observed between these studies. SPEM is thought to play a role in gastric epithelial healing, with studies showing induction of SPEM in regenerating stomach ulcers.^43^ Furthermore, epithelial regeneration in gastric ulcer disease is initiated by gastrin release,^44^ demonstrating the importance of G‐cells in this context. However, it should also be noted that previous studies suggesting that PD‐L1 expression in gastric epithelial cells may be induced upon *H. pylori* infection were mostly reliant on cancer cell lines (AGS) and mouse models investigated after acute *H. pylori* exposure, while our population consisted of patients that had already developed various degrees of GIM indicating a fairly long‐term colonization of the gastric epithelium with *H. pylori*.^25^, ^26^ In chronic infections, antigen‐presenting cells such as macrophages and dendritic cells, rather than epithelial or enteroendocrine cells, tend to express PD‐L1.^45^ Indeed, this is in line with our analysis of single‐cell datasets indicating that macrophages are the main immune cell population expressing PD‐L1 in gastric mucosa. The fact that we did not observe co‐localization of CD45 and PD‐L1 in our immunofluorescence staining may be caused by a scarcity of macrophages and other immune cells in these tissues.

*H. pylori* infection reduces the cell density of enteroendocrine cells in the gastric mucosa of GIM patients, as has been described before for otherwise healthy controls.^32^, ^46^, ^47^ However, this study shows that even in patients who have already developed GIM, eradication of *H. pylori* allows restoration of the enteroendocrine compartments to cell densities that are similar to those in patients that have never been infected with this bacterium. This underlines the recommendation of most clinical guidelines that eradication should be achieved, despite GIM being considered a ‘point of no return’ in the carcinogenic cascade.^48^ Furthermore, our study demonstrates that in the presence of persistent *H. pylori*, the percentage of G‐cells positive for PD‐L1 increases, suggesting that the gastric G‐cell compartment uses PD‐L1 signaling to escape *H. pylori*‐induced inflammatory destruction. It is unclear why, of all nonimmune cells, PD‐L1 expression is limited to G‐cells, as the expression of this gene has been shown to be induced in various cell types, in particular upon inflammatory signaling. This suggests that G‐cells may be particularly vulnerable to inflammatory environments, thus requiring specific protection. Indeed, it has been suggested that gastrin production by G‐cells is deregulated under inflammatory conditions.^49^, ^50^ This is exemplified by the decrease of G‐cells in patients infected with *H. pylori* as demonstrated in this study, and past research.^32^, ^46^, ^47^ As G‐cells serve an important role in the homeostasis of gastric acidity, in addition to playing an important role in the differentiation and maturation of the gastric epithelium, the selective rescue of G‐cell rescue under inflammatory conditions may be of particular benefit.^17^, ^51^ How the gastric antrum achieves this highly restrictive upregulation of PD‐L1 expression in just a singular cell type needs to be further investigated. Interestingly, recent studies revealed that other types of endocrine cells in normal endocrine tissues including the pancreas do not express PD‐L1 at significant levels,^52^ which would suggest that PD‐L1 is indeed specific to the antral G‐cell compartment.

In conclusion, we demonstrate that PD‐L1 is not expressed in GIM and thus is unlikely to play a role in GC progression from GIM. However, we show that antral G‐cells do express PD‐L1, resulting in protection from destruction during *H. pylori* infection. The constitutive expression of PD‐L1 in G‐cells in the gastric antrum in the presence of intestinal metaplasia may explain the occurrence of upper GI immune‐related adverse events in immunotherapy such as acute esophagitis gastritis or duodenitis.^53^, ^54^, ^55^ Indeed in the largest cohort study to date on these rare adverse events, the stomach was more often affected than the esophagus or duodenum.^56^ Our research may suggest a possible therapeutic use of PD‐1/PD‐L1 inhibition in Zollinger Ellison syndrome patients with either primary gastrinomas or other gastrin‐producing tumors.^57^, ^58^ However further research is necessary to see whether these tumors show the same phenotype as we found in the G‐cells of the gastric mucosa.

## AUTHOR CONTRIBUTIONS

MCM and GMF had full access to all study data and are responsible for data integrity and accuracy of the data analysis. The study concept and design were prepared by MCM, MPP, MD, JT, and GF. Acquisition of data was performed by MCM, BY, MD, and TPPB. Analysis and interpretation of data were performed by all co‐authors. Drafting of the manuscript was done by MCM and GMF. Critical revision of the manuscript for important intellectual content was performed by all authors. Statistical analysis was performed by MCM. Funding was obtained by MCWS, MPP, and GMF. Administrative, technical, or material support was provided by BY, MD, and TPPB. Study supervision was done by MPP and GMF.

## CONFLICT OF INTEREST

The authors have no potential conflicts of interest to disclose that are relevant to this manuscript. Full disclosures have been submitted to the journal.

## Supporting information


Appendix S1
