# Supplementary material for: Constitutive programmed death ligand 1 expression protects gastric G‐cells from Helicobacter pylori–induced inflammation
Source: Helicobacter. 2022 Jul 28;27(5):e12917. doi: 10.1111/hel.12917 (PMC9542424; doi:10.1111/hel.12917)
Supplement: Supplementary file 1 — Appendix S1 [file HEL-27-e12917-s001.docx]

Supplementary material belonging to

## Constitutive PD-L1 expression protects gastric G-cells from *H. pylori*-induced inflammation

M.C. Mommersteeg^1^, B. Yu^1^, T.P.P van den Bosch^2^, J.H. van der Thusen^2^, M. Doukas^2^, M.C.W. Spaander^1^, M. P. Peppelenbosch^1^, G.M. Fuhler^1^

**Supplementary tables**

|  | Dilution | Clone | Species | Company |
| --- | --- | --- | --- | --- |
| Gastrin IHC | 1:6 | polyclonal | rabbit | Leica Biosystems (Wetzlar, Germany) |
| Gastrin IF | 3.9ug/ml | polyclonal | mouse | Cell Marque (Rocklin USA) |
| Somatostatin IHC | 1:10.000 | polyclonal | rabbit | Phoenix pharmaceuticals (Burlingame, USA) |
| Somatostatin IF | 1: 400 | polyclonal | rabbit | DAKO (Santa Clara, USA) |
| Chromogranin A | 1.00ug/ml | LK2H10 | mouse | Roche (Basel, Switzerland) |
| CD45 | 4.14ug/ml | LCA | mouse | Cell Marque (Rocklin USA) |
| PD-L1 | 1.61ug/ml | SP263 | rabbit | Roche (Basel, Switzerland) |
|  |  |  |  |  |

**Supplementary Table 1.** Antibodies used in the study. IHC: immunohistochemistry. IF: immunofluorescence.

| Cell type | Marker genes |
| --- | --- |
| Gland mucous cell | MUC6, TFF2 |
| Pit mucous cell | MUC5AC, TFF1 |
| Enteroendocrine cell | CHGB, CHGA |
| Chief cell | PGA3, PGA4 |
| Proliferative cells | MKI67, TOP2A, BIRC5 |
| Goblet cells | MUC2, ITLN1, SPINK4 |
| Enterocytes | APOA1, ALPI, FABP1, APOA4 |
| T cells | CD2, CD3D |
| B cells | CD79A |
| Macrophage | CSF1R, CD68 |
| Fibroblasts | DCN, PDPN |
| Smooth muscle cells | ACTA2 |
| Endothelial cells | VWF, ENG |
| Mast cell | TPSAB1 |
| G cell | GAST, CHGA, CHGB |
| D cell | SST, CHGA,CHGB |

**Supplementary Table 2.** The known markers for cell lineages in stomach.

**Supplementary figures**

###
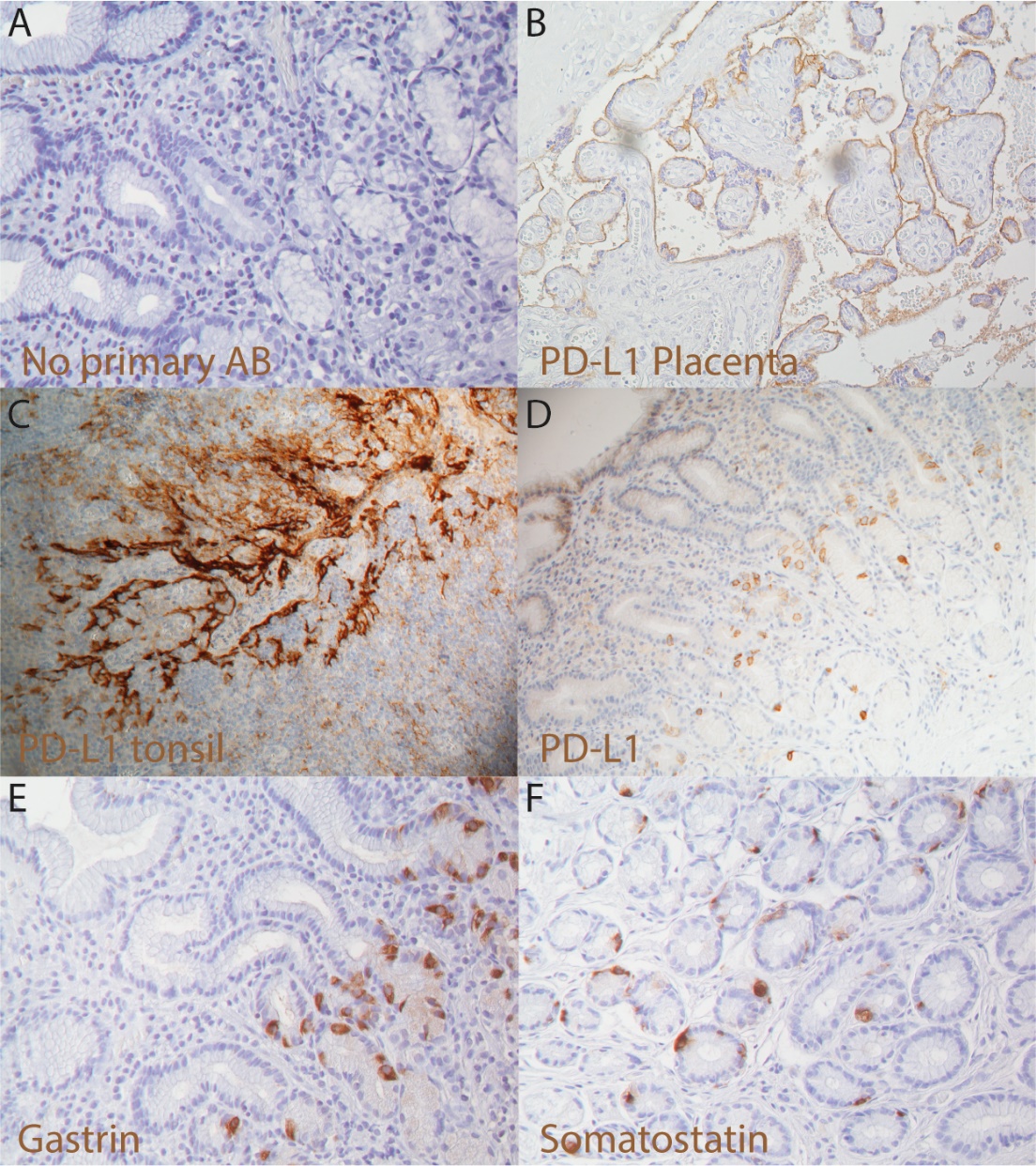


**Supplementary Fig. 1** Immunohistochemistry controls. (**A)** Antral biopsy, negative control for secondary antibody (no primary antibody added to slide). **(B, C)** Positive controls for PD-L1-SP263, respectively placenta and tonsil tissues. **(D)** Normal antral biopsy stained immunohistochemistry for PD-L1. **(E)** Normal antral mucosa stained for gastrin. **(F)** Normal antral mucosa stained for somatostatin.


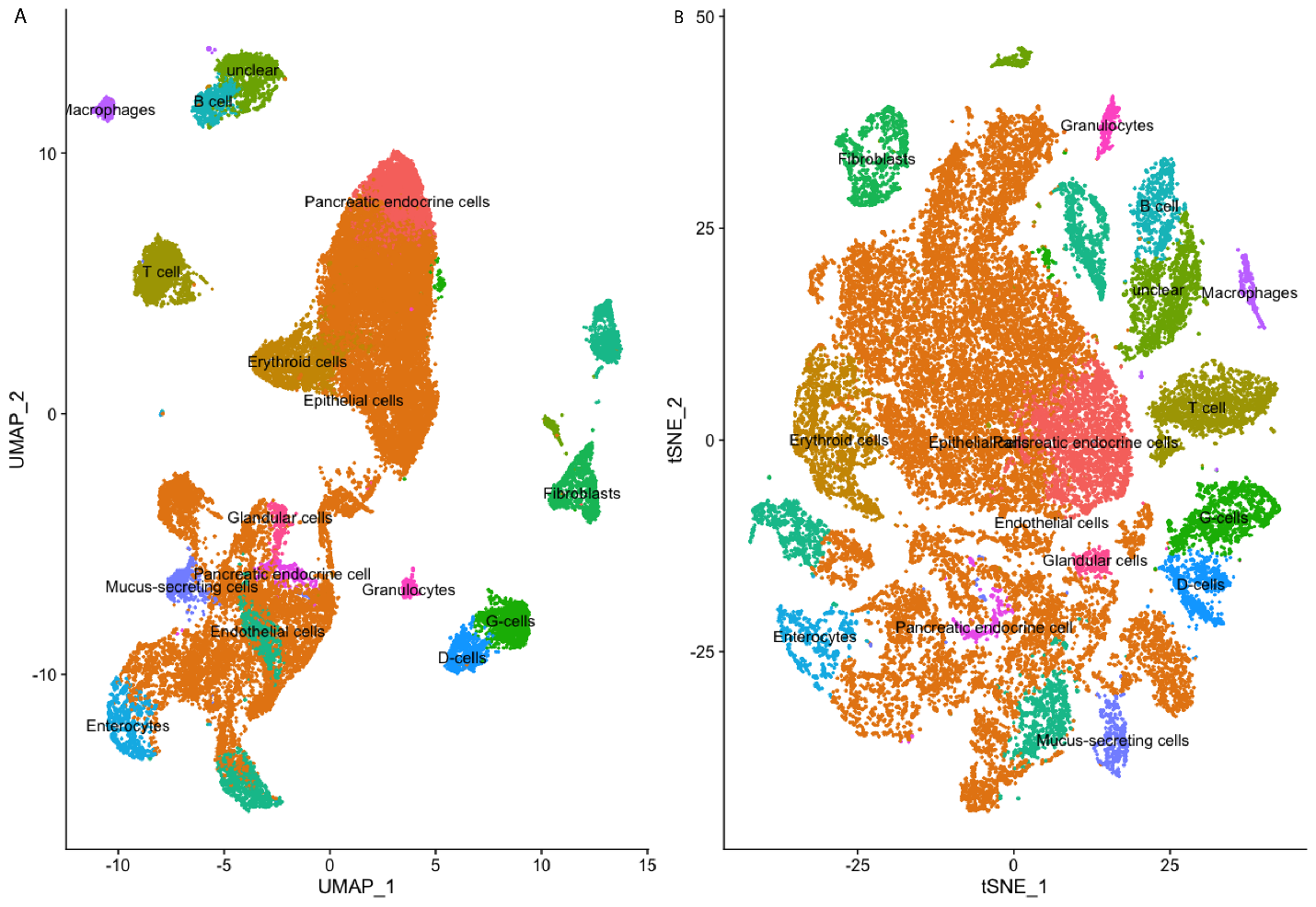


**Supplementary Fig. 2 A:** Uniform Manifold Approximation and Projection for Dimension (UMAP) plots for the 27677 high quality cells showing cell types. **B:** t Stochastic neighbour Embedding (tSNE) plots for the 27 677 high-quality cells showing cell types.

**Supplementary Fig. 3 No significant difference on the amount of PD-L1 positive cells in patients with progression or regression of GIM.** Quantification of the relative number of PD-L1 positive cells per high power of patients that will have progression or regression of their GIM (based on the operative link on gastric intestinal metaplasia assessment (OLGIM)) compared against patients with stable disease (dotted line.

###
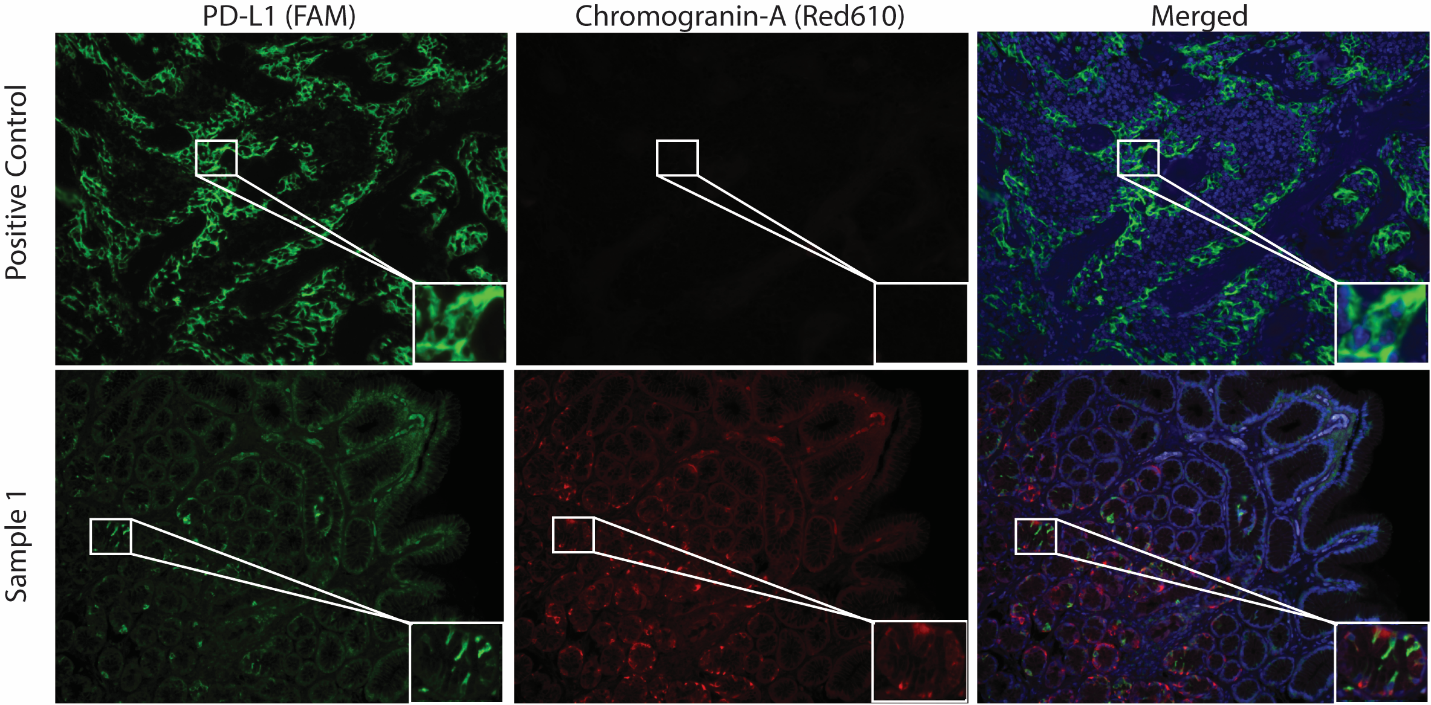


**Supplementary Figure 4**. **Multiplex immunofluorescence staining for PD-L1 (green) and chromogranin A (red).** Top row: positive control for PD-L1 (tonsilla tissue). Bottom row: an antral biopsy of an IM patient.


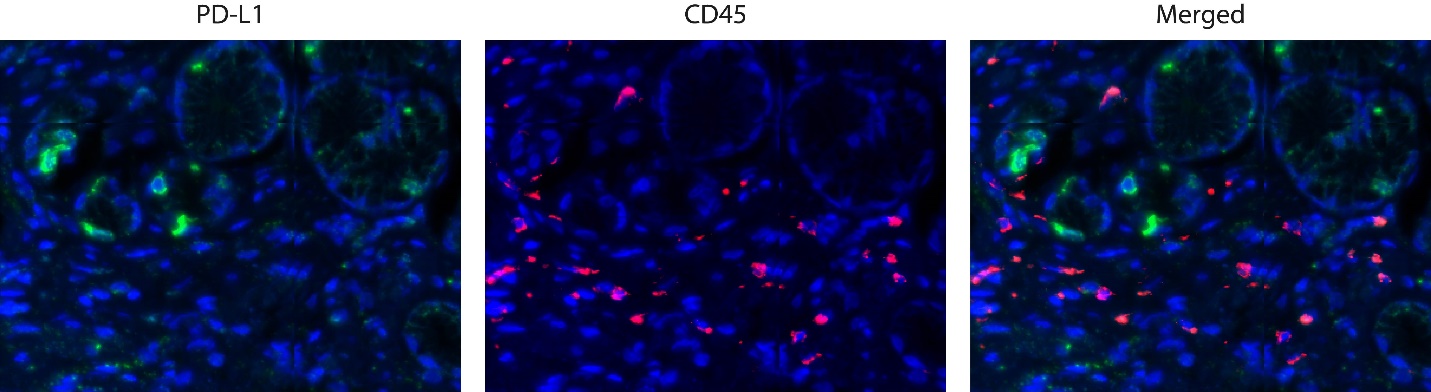


**Supplementary Fig. 5 Multiplex immunofluorescence staining for PD-L1 (green) and CD45 (red).** Antral biopsy showing CD45 positive cells mainly in the lamina propria.


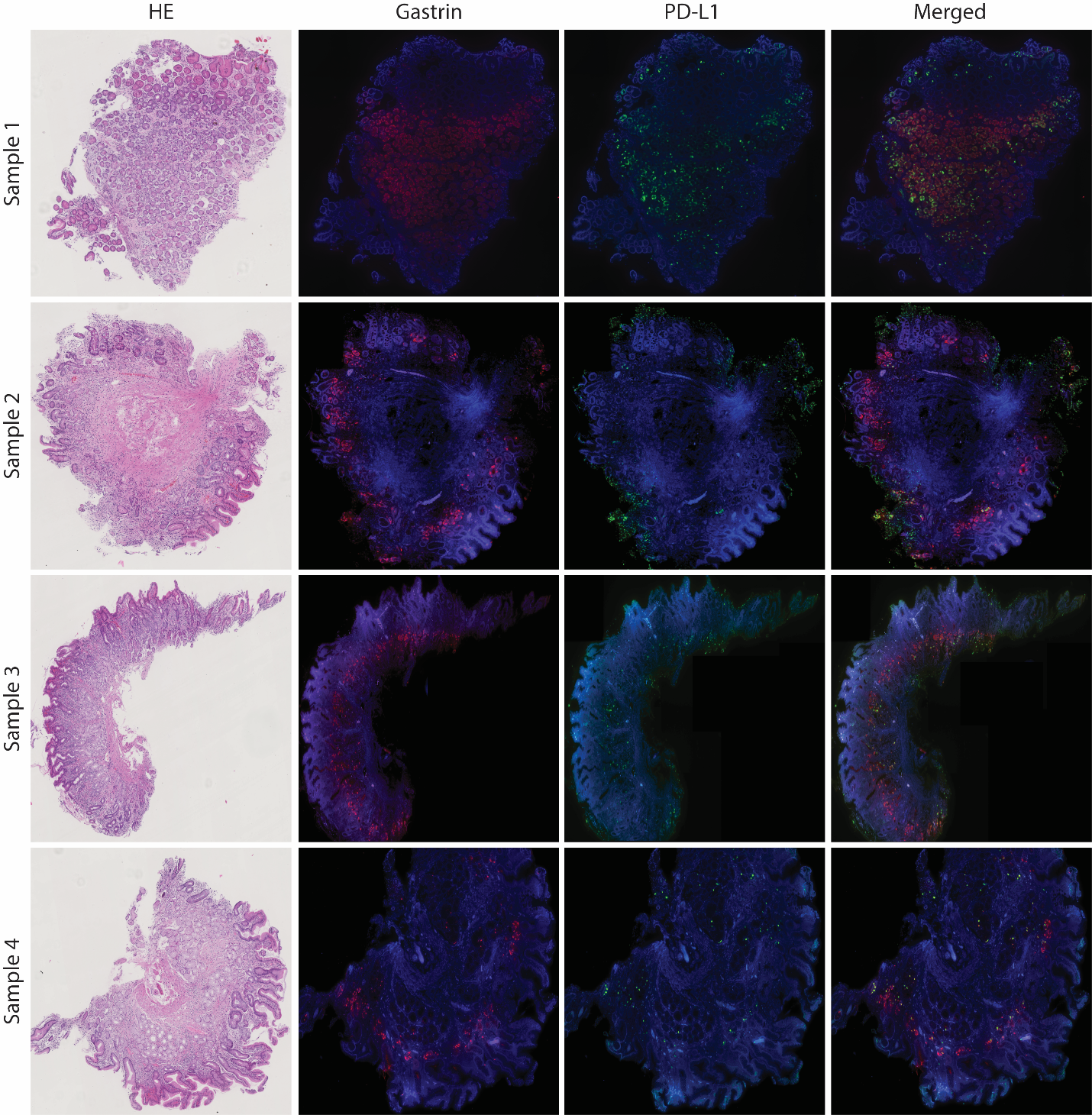


**Supplementary Fig. 6** **Overview images of gastric biopsies.** Four different complete gastric biopsies from four individual patients showing Hematoxylin and eosin stains (left panels) as well as multiplex immunofluorescence staining for PD-L1 (green), gastrin (red) and overlay.


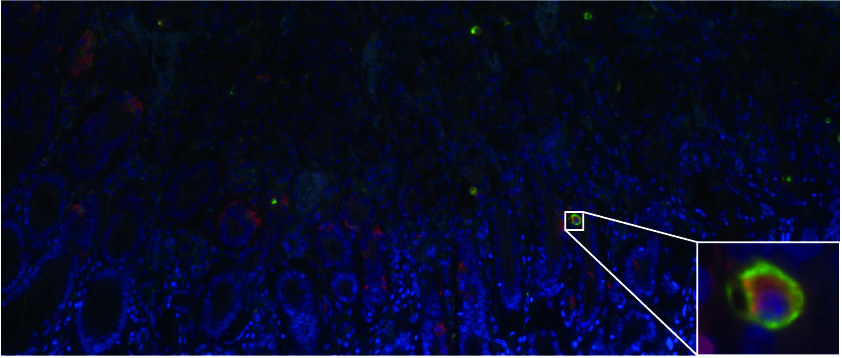


**Supplementary Fig. 7 PDL-1-positive G-cells present in normal stomach.** Gastric sleeve biopsy from a patient with no abnormalities nor *H. pylori* infection. Overlay shown from multiplex immunofluorescence staining for gastrin (red) and PD-L1 (green).
